# The effectiveness of adjusting resistance training loads through velocity-based techniques in experienced sprinters: a case series study

**DOI:** 10.3389/fphys.2023.1241459

**Published:** 2023-07-26

**Authors:** Violeta Muñoz de la Cruz, Aarón Agudo-Ortega, Vincenzo Sorgente, Anthony P. Turner, José María González-Ravé

**Affiliations:** ^1^ Sports Training Laboratory, Faculty of Sports Sciences, University of Castilla La Mancha, Toledo, Spain; ^2^ Kinesiology and Motor Control (Ki Mo Co) Laboratory, Department of Experimental and Clinical Medicine, Physiological Sciences Section, University of Florence, Florence, Italy; ^3^ Sport Physical Education and Health Sciences, University of Edinburgh, Edinburgh, United Kingdom

**Keywords:** resistance training, squat, track and field, individualization, performance

## Abstract

**Introduction:** This study aimed to determine if adjusting the loads via velocity-based training (VBT) in each session is more efficient in monitoring the relative intensity than programming loads assessing 1RM pre-training.

**Methods:** To achieve this, six national level sprinters were randomly divided into two groups, i.e., adjusting loads (AL, *n* = 3) and not adjusting loads (NAL, *n* = 3), during twelve sessions of a squat training (ST) program. During this training intervention, the AL group adjusted the intensity for each session in the squat exercise depending on the speed the load was lifted after warmup. The NAL group, instead, progressed in the squat exercise referring to the 1RM estimated at pre-test. In addition, Parallel Squat (PSQ), Countermovement Jump (CMJ), Squat Jump (SJ), 30 m sprint standing start (30S) and 30 m sprint flying start (30F) tests were carried out before and after conducting the ST program.

**Results:** Interestingly, AL performed the ST near their estimated velocities at 70%—75% 1RM, however with a wider gap at 80%—85% 1RM. The NAL group, instead, did not presented such a detectable behaviour across the whole ST. Moreover, both groups demonstrated improved performances in PSQ, CMJ, and SJ, whereas there were little changes in 30S and 30F after ST. Additionally, AL obtained a greater effect size than NAL in PSQ (0.60 vs. 0.35) but lower effect size in CMJ, SJ, 30S, and 30F (0.41 vs. 0.63, 0.30 vs. 0.40, 0.04 vs. 0.28 and 0.22 vs. 0.24). However, percentage change was greater in AL in all tests.

**Discussion:** Based on these findings, we can conclude that further investigation into the AL strategy in VBT is warranted for sprinter athletes’ daily strength practices. The AL technique shows promise as a valuable tool for accurately adjusting and monitoring medium-high training loads to ensure they align with the intended intensity.

## Introduction

Resistance training (RT) is commonly used for improving athletic performance in sprinters (Haugen et al., 2019). In particular, sprinters need to develop three key determinants: power, technique and sprint-specific endurance, as RT plays a paramount role in enhancing these neuromotor abilities (Moore, 2016; Haugen et al., 2019). Traditionally, the intensity during RT has mainly been prescribed using the percentage of one repetition maximum (%1RM), known as percentage-based-training (PBT) (Thompson et al., 2020).

With the %1RM approach, however, it is not possible to maintain the relative intensity (the movement execution velocity) throughout the RT session, e.g., from the first to the last set of an exercise. This is due to accumulated neuromuscular fatigue, associated overload, and possible muscle failure, eventually leading to an abrupt cessation of the set (González Badillo et al., 2017). In addition, the actual 1RM of an athlete can fluctuate in a relatively short time because of several intrinsic and extrinsic factors (González Badillo et al., 2017) (González Badillo et al., 2017). Research have also strongly advised against frequently testing 1RM to solve this issue, seeing that there are many feasibility complications with this practice, especially across multiple lifts (Pareja-Blanco et al., 2017).

To overcome these critical aspects, an alternative methodology known as velocity-based training (VBT) has been developed (Rodríguez-Rosell et al., 2020; Pelland et al., 2022; Zhang et al., 2022). VBT consists in monitoring the speed at which the load is lifted using a linear position transducer (LPT) and then estimating the 1RM (González-Badillo and Sánchez-Medina, 2010) This is possible thanks to the relationship between load and velocity, where the higher the load, the lower the execution velocity (González-Badillo and Sánchez-Medina, 2010). In this regard, it has been shown that each strength exercise has specific 1RM lifting speeds, e.g., the 1RM of a full squat would be at a speed of 0.32 m per seconds, although this may vary slightly between individuals (Sánchez-Medina et al., 2017). Therefore, this technique allows measuring the daily readiness and the decrease of velocity which represents the accumulation of fatigue, being less susceptible to changes than the %1RM method (González-Badillo and Sánchez-Medina, 2010). Moreover, VBT has shown greater effects on improving high-speed actions such as sprinting or countermovement jump (CMJ), compared to traditional RT like circuit training (Dorrell et al., 2019; Banyard et al., 2021). In fact, a systematic review about this approach concluded that VBT could be recommended as a useful tool in terms of obtaining instantaneous objective feedback, as it provides velocity data during the training (Włodarczyk et al., 2021). Although VBT is increasingly used in research and by athletes’ coaches (Suchomel et al., 2021), there is a research gap in the way in which we can monitor the daily relative intensity through velocity of execution and efficiently account for the daily changes in the athletes’ performance capabilities. In fact, there is currently no evidence to support whether adjusting loads and monitoring intensity during RT programs improves power/velocity abilities in trained sprinters, who usually combine strength training with their track training, compared to not adjusting loads each session. To this matter, while it has been shown that adjusting loads according to speed performances produces no greater improvement than not adjusting the load in full squat after 8 weeks of training (Jiménez-Reyes et al., 2021), there are some critical aspects about this approach, i.e., not considering relative volume (measured by percentage of velocity loss) as they programmed establishing the same sets and repetitions for all participants. The population tested should be taken into account as they were students with RT experience and not highly trained athletes.

With these considerations in mind, in this case-series study we sought to compare the effectiveness of two VBT approaches in monitoring relative intensity across 6 weeks (twelve sessions) of squat training (ST) intervention in a group of six highly trained sprinters. In particular, one VBT strategy consisted in adjusting the load for each of the ST session, whereas the other one consisted in establishing the 1RM load at pre-test and conducting a traditional load progression accordingly. In addition, we sought to compare the effects of these VBT strategies in improving fundamental aspects of athletic performance, testing for markers of maximal strength and jumping pre- and post-ST, such as parallel squat (PSQ), countermovement jump (CMJ), squat jump (SJ), 30 m sprint standing start (30S) and 30 m sprint flying start (30F). Ultimately, this study would contribute to the existing body of knowledge with preliminary insights about which kind of VBT to use when striving to enhance the competitive form of track and field practitioners.

## Materials and methods

### Participants

Six track and field sprinters from the same training group were included in this study. All participants were Caucasian male from the regional team of Castilla La Mancha (Spain) (Age 20 ± 1 years; body mass 70.2 ± 3.2 kg, leg length 78.3 ± 3.1 cm and height 175.9 ± 5.7 cm) with a mean of 844.2 ± 88.6 points in their best discipline according to the World Athletics Federation scoring tables, considering both outdoor and indoor times. Hence, they were classified as level 3: Highly trained/National according to McKay et al. (2022). The subjects had experience with the squat exercise and more generally in resistance training. They had more than 5 years of resistance training experience and had competed in sprint events for more than 5 years as well. However, they had never trained with exerting the highest possible speed as training goal and they were not in their life’s peak of performance as normally sprinters achieve it at an age of 25–27 (Haugen et al., 2018). The participants were divided into two groups, adjusting load (AL, *n* = 3) and not-adjusting load (NAL, *n* = 3). The study design followed the ethical principles for medical research involving human participants set by the World Medical Association Declaration of Helsinki and was approved by the local ethics committee. Furthermore, participants were provided with written instructions outlining the procedures and risks associated with the study and gave informed written consent.

### Experimental design

The experiment was conducted over a duration of 9 weeks. Prior to the pre-tests, the participants underwent a 1-week familiarization period with Velocity-Based Training (VBT). During this period, they engaged in two brief sessions where they practiced lifting light loads in the squat exercise with the goal of lifting them as quickly as possible. This familiarization phase aimed to acclimate the participants to the VBT technique. Then, two pre-test sessions were carried out. In particular, performances in PSQ, CMJ, SJ, 30F, and 30S were assessed. In the first pre-test session, participants performed CMJ and PSQ respectively. During the second pre-test session, performances in SJ, 30S, and 30F tests were evaluated. After the pre-test, the ST intervention was conducted. The ST consisted in 6 weeks (two sessions per week) of the same programme for both groups. However, the programme differed in how the load was managed during each session. That is, in the AL group, the daily squat load was readjusted from the velocity performed during the 1RM pre-test. This readjustment was performed at the end of the warmup phase (Jiménez-Reyes et al., 2021). Regarding the NAL group, the squat load was measured at the baseline and the load progression was designed accordingly, thus without further adjustments. Finally, post-test evaluations were conducted in the same manner as the pre-test phase.

The independent variable in this research was the relative intensity, indicated by the exercise execution velocity measured in m/s for each session according to previous studies (Jiménez-Reyes et al., 2021). The experimental design is further illustrated in Table 1.

**TABLE 1:** Experimental design of the study.

Phase	Familiarization	Pre-test		Squat training*				Post-test	
Activity	VBT with light loads	PSQ & CMJ	SJ, 30S, & 30F	70%	75%	80%	85%	PSQ & CMJ	SJ, 30S, & 30F
				3 sets until detecting 15% in VL					
				4′ rest between sets					
# of sessions	2	1	1	3	3	3	3	1	1
Duration	1 week	1 week (two total sessions)		6 weeks (twelve total sessions)				1 week (two total sessions)	

Legend: VBT, velocity based training; PSQ, parallel squat (kg); CMJ, counter movement jump (cm); SJ, squat jump (kg); 30S, 30 m standing start (s); 30F, 30 m flying start (s). VL, velocity loss; *, Training loads referring to the 1RM%.

### Assessment of the relative intensity in the squat training

To measure the velocity in the ST, the LPT (Chronojump; Boscosystem, Barcelona, Spain) was employed.

During the ST, both groups warmed up performing the squat exercise with submaximal loads, i.e., one set with 40% 1RM and one set with 60% 1RM, evaluated using the LPT. The set was finished when we detected 15% in VL. After 5 min of rest from this warmup sets, we proceeded with the squat velocity evaluation. Specifically, subjects performed two repetitions with the programmed load estimated at pre-test. Regarding the AL group, if performed velocity differed more than 0.05 m·s^−1^ from the programmed one, the training load for the subsequent ST was readjusted of ±5 Kg.

### Squat training programme

The squat training programme consisted of 12 sessions across 6 weeks (two sessions per week). In terms of intensity training progression, participants started performing 70% of estimated 1RM and incremented by 5% every three sessions until 85% estimated 1RM. All sessions consisted in 3 sets of squats recovering 4’ between sets. Participants performed the set at the maximal intentional velocity. The set was stopped when two consecutive repetitions were performed at a velocity slower than 15% from the fastest repetition, which was generally the first or second repetitions of the set. This means we did not considered repetitions to monitor volume. All strength sessions were supervised by a strength and conditioning coach and co-author of this article. Apart from ST, participants performed their usual track and field sessions, supervised by their coach, as they were preparing the indoor season. Nevertheless, they were not in a competition period, thus workouts were general and the same for all of them.

In order to avoid fatigue and metabolic stress produced during the regular track and field workouts, the ST was always conducted at least 8 h before.

### Pre-post evaluation

The protocols adopted for CMJ, PSQ, and SJ were those proposed by Bachero-Mena et al. (2019), whereas the protocols for 30S and 30F were adapted from (Loturco et al. (2015a). Regarding CMJ, 10 submaximal jumps were performed to warm up. After 1 min of rest, 3 maximal jumps were then carried out recovering 1 min. The average height (cm) of these three jumps was considered for analyses purposes. Jump height was measured using Optojump (Microgate, Bolzano, Italy). PSQ incremental test consisted of lifting incremental loads from 20 Kg and adding 10 Kg until a execution velocity of 0.45 m∙s^−1^ when the test ended. Three repetitions per load were conducted when the velocity was higher than 1.15 m∙s^−1^, when the movement velocity was slower than 0.7 m∙s^−1^ two repetitions were performed and when it was slower than 0.5 m∙s^−1^ the participants only lifted the weight once per set. The velocity in the PSQ incremental test was measured through a linear encoder (Chronojump, Boscosystem, Barcerlona, Spain). Moreover, we employed the PSQ incremental test to develop the force-velocity profile of each participant. By leveraging on the well-known load-velocity relation (which is *R* ^2^ > 0.98) (Samozino et al., 2012; González Badillo et al., 2017; Sánchez-Medina et al., 2017; Sorgente et al., 2023), from the force-velocity profile we were able to establish the load intensity for each ST session. At least six loads were needed, which represent points in the linear relationship As for the SJ test, participants started the exercise with only their bodyweight, progressively increasing the load by 5-10 Kg until they jumped as low as 20 cm, which represents the optimum mean propulsive power of the athlete (Loturco et al., 2015b). Participants carried out two repetitions per load (we considered the highest for posterior analyses) and recovered 3 min between sets. As in CMJ test, jump height was measure using Optojump system (Microgate, Italy, Bolzano). Finally, the 30S and 30F tests were performed twice (two sets), recovering 3 min between sets, and considering the best sprint time of each respective test for subsequent analyses. Sprints were conducted in the same athletics track for each test, in similar weather and wind conditions and using the same spikes. A photocell timing system (Witty tireless timing system, Microgate, Bolzano, Italy) was used for recording the sprinting times. Both photocells were placed at the same height in all the tests performed by the different participants (60 cm) as the placement of the photocells at different heights can cause variations in the times recorded (Cronin and Templeton, 2008). To avoid interference of one test in another test, Pre and post-tests were performed in the following distribution: Day 1: CMJ–PSQ and day 2: SJ, 30S and 30F.

### Data analyses

**FIGURE 1:**
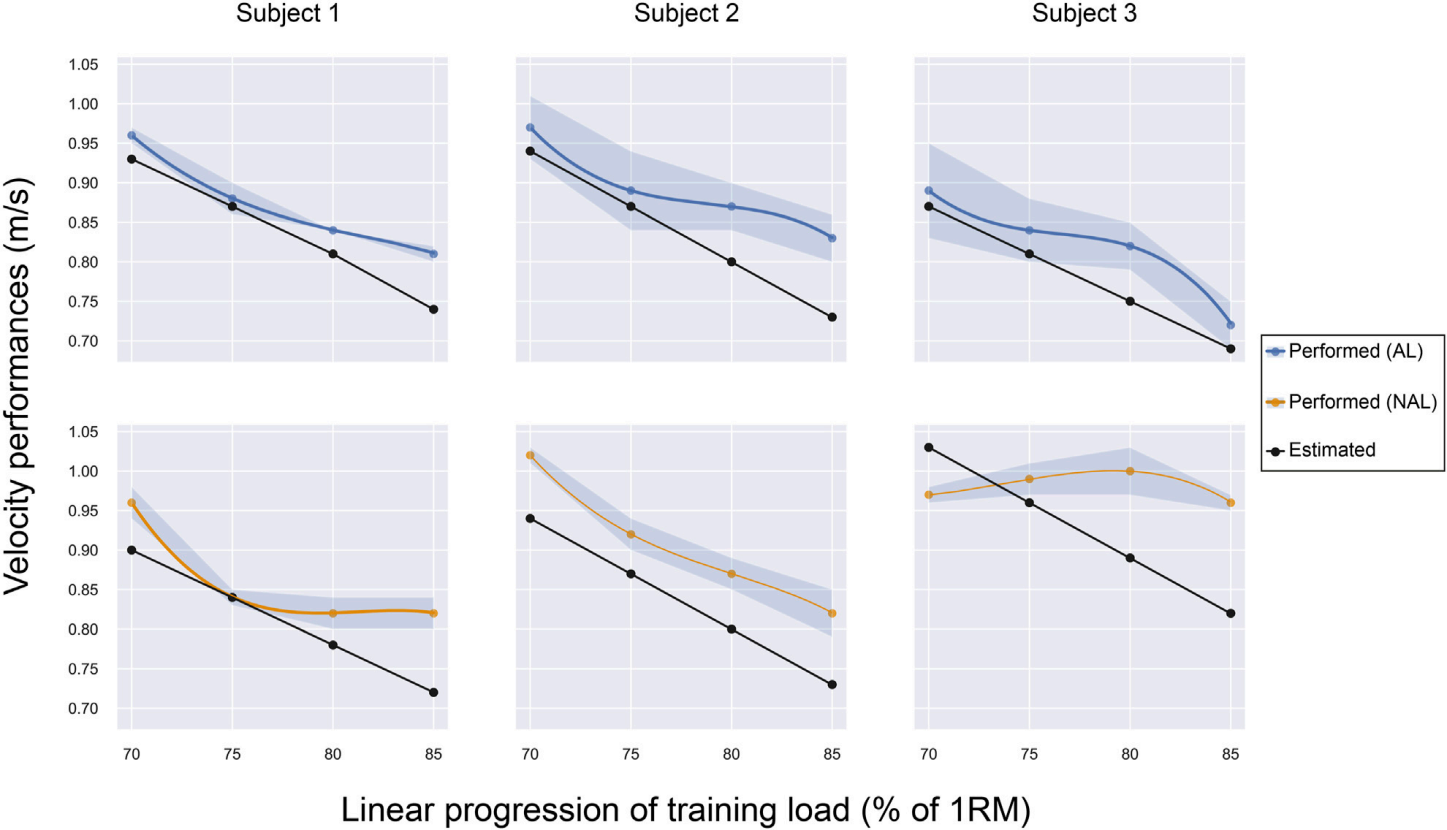
Regression plot showing the estimated vs. performed velocity progression in ST for each subject. The coloured points representing the two groups (blue for the AL group, orange for the NAL group) refer to the average peak of velocities obtained during the ST at that specific percentage of 1RM.

Data were presented as mean ± standard deviation. Due to the small sample size, we considered the value of the effect size as measured by Cohen’s d, and the percentage change (PC) between pre and post-tests in both groups. This is because the thorough application of inferential statistics, e.g., *t*-test or ANOVA, can be misleading in this type of sample where differences between groups at the *p* < 0.05 level may not be properly identified (Rhea, 2004). However, to ensure no differences at the baseline among groups, we conducted a Mann-Whitney *U*-test for independent variables comparing AL and NAL group performances in the pre-test battery. Descriptive data were showed about the estimated vs. performed velocity progression in ST for each subject in the Figure 1. Analyses were done with Jamovi 2.2.5 for Windows.

## Results

The Mann-Whitney *U*-test indicated that there was no significant difference between the pre-test performances of the AL and NAL groups, *U* = 5, *p* = 1 for all the pre-test battery. This showed that the two groups were homogeneous in terms of squatting, jumping, and running performances at the baseline. Moreover, the post-test analysis showed that the PC in squatting (PSQ) and jumping (CMJ and SJ) performances were all positive, signalling that the participants enhanced their expressions of vertical force abilities after the concurrent ST and usual track and field training. Furthermore, these changes were greater in the AL group compared to the NAL group. However, regarding the running performances (30S and 30F tests), the NAL group obtained a small, negative percentage change (0.65%) in the 30S test, whereas the AL group performed minimally better than pre-test (−0.16%). Besides, both groups slightly improved their performance in the 30F post-test (−1.98% and −1.10%, respectively). The Cohen’s d ranged from very small to medium effect sizes. In particular, the effect sizes were the highest in squatting and jumping performances for both groups, reporting medium effects for the PSQ (0.60 in the AL group) and CMJ (0.62 in the NAL group). Additionally, the AL group presented higher effect size in the PSQ (0.60 vs. 0.35 in the NAL group), whereas the NAL group had the higher effects in CMJ (0.63 vs. 0.41 in the AL group) and SJ (0.40 vs. 0.30 in the AL group). On the contrary, the smallest effects concerned the running performances, ranging from 0.04 for the 30S in the AL group, to 0.28 for the same test in the NAL group (Table 2).

**TABLE 2:** Results from the pre and post-test battery. For both groups, the percentage changes and effect sizes were the highest when considering squatting and vertical jumping performance, i.e., in the PSQ, CMJ, and SJ tests.

AL					NAL			
	Pre-test	Post-test	PC	d	Pre-test	Post-test	PC	d
PSQ (kg)	115.31 ± 27.80	131.97 ± 36.64	14.45	0.60	108.83 ± 27.57	118.57 ± 14.92	8.96	0.35
CMJ (cm)	38.67 ± 9.21	42.47 ± 9.91	9.83	0.41	43.40 ± 5.16	46.63 ± 9.54	7.45	0.63
SJ (kg)	30.33 ± 17.47	37.00 ± 23.07	21.98	0.30	32.83 ± 15.94	39.33 ± 14.36	19.80	0.40
30S (s)	4.14 ± 0.16	4.13 ± 0.21	−0.16	0.04	4.11 ± 0.10	4.14 ± 0.18	0.65	0.28
30F (s)	3.37 ± 0.30	3.30 ± 0.29	−1.98	0.22	3.34 ± 0.15	3.30 ± 0.16	−1.10	0.24

Legend: AL, adjusting loads group; NAL, not adjusting loads group; PC, percentage change; d, Cohen’s d effect size; PSQ, parallel squat (kg); CMJ, counter movement jump (cm); SJ, squat jump (kg); 30S, 30 m standing start (s); 30F, 30 m flying start (s).

Interestingly, the outcomes from the ST revealed a particular trend regarding the AL group (Table 3). Specifically, the difference between the performed and estimated velocities in this group was positive across the entire ST. This meant that the athletes from the AL group systematically performed at higher velocities than the ones we estimated via F-V profiling.

**TABLE 3:** Progressions of the performed velocity-based ST compared to the estimated one. Performed velocity is reported as the mean of the velocity peaks performed in each session which employed a specific percentage of 1RM.

Group/subject	% of RM	70%	75%	80%	85%
AL, S1	P	0.96 ± 0.01	0.88 ± 0.02	0.84 ± 0.00	0.81 ± 0.01
	E	0.93	0.87	0.81	0.74
AL, S2	P	0.97 ± 0.04	0.89 ± 0.05	0.87 ± 0.03	0.83 ± 0.03
	E	0.94	0.87	0.80	0.73
AL, S3	P	0.89 ± 0.06	0.84 ± 0.04	0.82 ± 0.03	0.72 ± 0.03
	E	0.87	0.81	0.75	0.69
NAL, S1	P	0.96 ± 0.02	0.84 ± 0.01	0.82 ± 0.02	0.81 ± 0.02
	E	0.90	0.84	0.78	0.72
NAL, S2	P	1.02 ± 0.01	0.92 ± 0.02	0.87 ± 0.02	0.82 ± 0.03
	E	0.94	0.87	0.80	0.73
NAL, S3	P	0.97 ± 0.01	0.99 ± 0.02	1.00 ± 0.03	0.96 ± 0.01
	E	1.03	0.96	0.89	0.82

Legend: AL, adjusting loads group; NAL, not adjusting loads group; S, subject; P, performed velocity (m/s); E, estimated velocity (m/s).

However, two distinct tendencies could be identified within the ST load progression. In particular, at 70%—75% of the predicted 1RM, the AL group performed at velocities close to the estimated values, with the difference between performed and estimated velocity ranging from to 0.01–0.03 m/s. Progressing through higher loads, i.e., at 80%—85% of the 1RM, the AL group tended to exceed the predicted velocities, with the difference between performed and estimated velocity rising up from 0.03 to 0.10 m/s (Figure 1).

The NAL group, on the other hand, showed a more heterogeneous behaviour. Namely, within the 70%–75% range of 1RM, the velocity performances could happen to be exactly the same, much higher, or even less than estimated, which was never the case regarding the AL group. Moreover, the differences between performed and estimated velocities were the highest in the NAL group, particularly at 80% and 85% of 1RM, accentuating the trend already shown within the AL group.

## Discussion

The aim of this study was twofold. One objective was to compare the effects of two different squat VBT strategies (AL vs. NAL) for enhancing maximal squatting, jumping, and sprinting performances in track and field athletes. Another intent was to explore whether one VBT strategy was more precise than the other in identifying training intensity mismatching between estimated and performed velocities.

We found that the AL strategy appears to be the most optimal tool within VBT to effectively control the relative intensity across a linear progression of training loads. This seems particularly efficient while training at medium intensities, i.e., 70% and 75% 1RM, whereas this effect is less accurate at higher intensities, i.e., 80% and 85% 1RM. We also found the AL strategy capable of better improving maximal squatting and vertical jumping performances, whereas very little performance changes were found in sprinting performances after the ST intervention.

Effects in physical performance produced by the AL vs. NAL approaches have been previously studied. For instance, Jiménez-Reyes et al. (2021) found that the NAL group obtained significantly greater results than the AL group in back squat 1RM, CMJ and sprint performances after the ST. The authors speculated that this phenomenon could be due to the use of lower loads than the scheduled ones across the ST. They concluded that a stimulus inducing low degrees of fatigue may be enough to elicit strength adaptations in a non-experienced population. Nevertheless, our results contrast with these findings, as we identified greater percentage changes in favour of the AL group in PSQ, CMJ, and SJ after the ST intervention. Nevertheless, NAL obtained greater ES than AL in all tests except from PSQ. This might be because NAL was training at higher velocities than AL (as the relative intensity was lower), working on fast movements, which are found in jumping and sprint actions. In fact, Rodríguez-Rosell et al. (2017), analysed the effects of light-load training and combined training including heavy loads and they found higher efficacy of transfer of strength gains to sprint ability in light-loads group.

It is also worth mentioning that the two sprinting tests we employed (30S and 30F) revealed minimal percentage changes and ES in both groups. Here, the nature of the population recruited, i.e., national track and field sprinters, could explain the lower effect in these tests. Given that short-distance repeats are a paramount activity for sprinters, our participants were most likely accustomed to sprinting from years of previous training. On the contrary, participants from Jiménez-Reyes et al. (2021) enhanced their performance in short distance sprinting. However, they were physically active men with less experience in sprinting executions, having more room for improving their athletic features than experienced sprinters.

Nonetheless, improvements in sprint performance are not assessed exclusively via outcome measurements, as the one we employed in this study (sprint time). For example, enhancement in sprinting can be analysed through biomechanical and kinematic factors, e.g., long ground contact times during the acceleration phase (Weyand et al., 2000), or great concentric force production of knee and hip flexors (Dorn et al., 2012). These factors are also achievable focusing on training and improving maximum strength levels (Cormie et al., 2010). Hence, although our participants did not improve their sprint times after the ST, it is still possible that there were improvements concerning other internal factors, which were not evaluated in this study. Remarkably, the AL and NAL groups showed different interactions between estimated and performed velocities. With respect to the AL group, there was a homogeneous trend throughout the ST program. Namely, the gap between the estimated and performed velocities was minimal at lower intensities (from 0.01 to 0.03 m/s at 70%—75% 1RM) Supporting this outcome. Jiménez-Reyes et al. (2021) also found minimal differences between estimated and performed velocity in the AL group. However, the gap in our experiment started to increase during the second half of the ST, where the intensity went progressively up (from 0.03 to 0.10 m/s at 80%—85% 1RM). Seeing that in the AL group the training load was adjusted in each session during the warmup sets, an inefficient warmup protocol could lead the athletes to lift lighter loads than they had to, thus setting a lower 1RM for that particular training session. Therefore, it may be the case that the protocol proposed could have not been enough, in terms of neural activation, to prepare the athletes to perform at high intensities such as 80%–85% 1RM. Perhaps, different timing and loading techniques for optimal neural activations could be used to this scope, e.g., PAP and/or PAPE, ramping techniques, etc.

On the other hand, regarding the NAL group, the estimated and performed velocities followed an inconsistent behaviour for every load percentage. That is, each participant presented a singular behaviour in terms of intensity progression, with peculiar cases of either matching exactly or performing below the estimated intensity. Despite this, the NAL group tended to perform at higher velocities than the estimated ones, with greater gaps compared to the AL group. Moreover, the gaps even widened as the sessions progressed. For instance, subject 3 followed the most mismatching progression and was training at the same relative intensity during the whole ST. This could be because his training adaptations appeared earlier than the load increment and therefore intensity progression. Training adaptations characteristics depend on a great variety of factors as molecular processes genetically predisposed, nutrition or acclimatization (Hughes et al., 2018). Therefore, training adaptations should be considered individually. In line with our study, Jiménez-Reyes et al. (2021) found significant differences between estimated and performed velocities from session 5 (65% 1RM) onwards in the NAL group.

The main limitation of this study is the small sample size, which may limit the generalizability of the findings. It is important to conduct further research with larger and more diverse samples to confirm the trends observed in this article. Additionally, the low statistical power resulting from only including six participants may lead to potential misinterpretation of critical results. Regardless, there is little scientific literature about this topic, thus a case series study can still be considered an initial piece of valuable information for coaches and scholars who seek to make their designed training loads adhere to the actual strength performances. Furthermore, case studies can serve as a useful communication channel with coaches and may develop hypotheses and effect sizes useful in designing further studies (Halperin, 2018).

## Conclusion

Adjusting ST intensity during VBT produced performance improvements in strength and sprint skills and additionally, holds potential to drive the in-field research towards more efficient training periodization and management throughout the track and field athletes’ competitive season. The trend of matching or mismatch between absolute load and relative intensity is observable and a more adequate control is obtained by using the daily load adjustment through velocity. This study provides promising information and preliminary recommendations for coaches who want to adjust the intensity of training to the athlete’s daily condition considering factors as internal fatigue.

## Data Availability

The original contributions presented in the study are included in the article/supplementary materials, further inquiries can be directed to the corresponding authors.
